# Phenome‐to‐genome insights for evaluating root system architecture in field studies of maize

**DOI:** 10.1002/tpg2.70100

**Published:** 2025-08-24

**Authors:** Kirsten M. Hein, Alexander E. Liu, Jack L. Mullen, Mon‐Ray Shao, Christopher N. Topp, John K. McKay

**Affiliations:** ^1^ Department of Soil and Crop Sciences Colorado State University Fort Collins Colorado USA; ^2^ Donald Danforth Plant Science Center Saint Louis Missouri USA; ^3^ Dümmen Orange De Lier The Netherlands

## Abstract

Understanding the genetic basis of root system architecture (RSA) in crops requires innovative approaches that enable both high‐throughput and precise phenotyping in field conditions. In this study, we evaluated multiple phenotyping and analytical frameworks for quantifying RSA in mature, field‐grown maize in three field experiments. We used forward and reverse genetic approaches to evaluate >1700 maize root crowns, including a diversity panel, a biparental mapping population, and maize mutant and wild‐type alleles at two known RSA genes, *DEEPER ROOTING 1* (*DRO1*) and *Rootless1* (*Rt1*). We show the utility of increasing the dimensionality of traditional two‐dimensional (2D) techniques, referred to as the “2D multi‐view” method, to improve the capture of whole root system information for mapping genetic variation influencing RSA. Comparison of univariate and multivariate genome‐wide association study (GWAS) approaches revealed that multivariate traits were effective at dissecting complex RSA phenotypes and identifying pleiotropic quantitative trait loci (QTLs). Overall, three‐dimensional (3D) root models generated from X‐ray computed tomography and digital phenotyping captured a larger proportion of RSA trait variations compared to other methods of root phenotyping, as evidenced by both genome‐wide and single‐gene analyses. Among the individual root traits, root pulling force emerged as a highly heritable estimate of RSA that identified the largest number of shared QTLs with 3D phenotypes. Our study shows that integrating complementary phenotyping technologies helps to provide a more comprehensive understanding of the genetic architecture of RSA in field‐grown maize.

## Core Ideas

- Complementary phenotyping approaches are powerful for studying the genetic basis of root system architecture.
- Multi‐view imaging enhances root trait estimation in two‐dimensional root crown phenotyping for genotype–phenotype mapping.
- Multivariate approaches effectively capture complex root traits and their underlying genetic architecture.
- Three‐dimensional phenotyping offers detailed insights to root crown analysis for forward and reverse genetic studies.
- Root pulling force is highly heritable and cost‐effective for large‐scale root crown field phenotyping.

## Plain Language Summary

Understanding the genetic and molecular mechanisms that regulate root system growth and function in plants is crucial for enhancing crop resilience to environmental stress and for promoting sustainable agriculture. However, the investigation of complex root system dynamics under field conditions remains limited by the challenges of large‐scale sampling and precise phenotyping. This study used forward and reverse genetics in three maize field experiments to assess different root phenotyping methods in capturing features of root crown phenome and identifying genotype–phenotype relationships. High‐dimensional phenotyping, including two‐dimensional multi‐view and three‐dimensional image analysis, combined with multivariate analysis, offered a more detailed understanding of the complex trait relationships and genetic architecture underlying maize root crowns. These findings underscore the value of integrating complementary approaches to bridge the phenotype‐to‐genotype gap in field studies of belowground traits.

**Abbreviations**
BLUP: best linear unbiased predictor
DIRT: digital imaging of root traits
*DRO1*
: *DEEPER ROOTING 1*
FarmCPU: fixed and random model circulating probability
GWAS: genome‐wide association study
MANOVA: multivariate analysis of variance
NAM: nested association mapping
PCA: principal component analysis
PVE: phenotypic variance explained
QTL: quantitative trait locus
RIL: recombinant inbred line
RPF: root pulling force
RSA: root system architecture
*Rt1*
: *Rootless1*
SAM: shoot apical meristem
SNP: single‐nucleotide polymorphism
SCD: steep, cheap, and deep
XRT: X‐ray tomography

## 1 INTRODUCTION

Root systems play critical roles in plant acclimation by modulating the timing and location of growth in response to water and nutrient availability. Genetic variation in root systems is also important for adaptation across species, germplasm, and environments. Such genetic variation in root traits can be utilized to achieve breeding targets for enhancing crop resiliency. Despite their importance in plant development and fitness, the quantitative genetic control of root traits, particularly in field‐grown crops, remains underexplored. This gap in understanding is primarily due to the difficulty of obtaining high‐quality measurements from hundreds or thousands of samples. With the emergence of various high‐throughput root phenotyping technologies in field environments, we investigate the relative value of these methods to expand our current understanding of the overall root system and its underlying genetic architecture.

Root system architecture (RSA) is a term that describes the spatial organization of root systems, integrating developmental, anatomical, and topological aspects of root–environment interactions (Fitter, 1987). Concerted efforts have been made to develop standardized terminology and classification schemes for root systems, led by the International Society of Root Research and Plant Ontology Consortium (Feix et al., 2002; Ilic et al., 2007; Zobel & Waisel, 2010). These classifications provide precise descriptions of root function and structure, accommodating the inherent complexity and plasticity of root systems, across species and environments. RSA demonstrates notable plasticity in response to environmental factors such as nutrient and water availability, soil composition, and rhizosphere biotic interactions (Clarke et al., 2019; Johnson & Rasmann, 2015; Karlova et al., 2021; Lareen et al., 2016). The regulation of specific root traits, such as root angle and crown root number, has been shown to improve mobile and immobile nutrient acquisition in response to different environmental scenarios (Bonser et al., 1996; de Jong et al., 2019; Gaudin et al., 2011; J. P. Lynch & Brown, 2001; Saengwilai et al., 2014; Sun et al., 2018; Trachsel et al., 2013; York & Lynch, 2015). By integrating systematic classifications with ongoing research, our understanding of RSA can facilitate innovative breeding strategies that optimize plant productivity and adaptation to specific production environments and management (Schneider & Lynch, 2020).

Root crowns are the nexus of the entire root system and are therefore information‐rich targets of RSA investigations. Advances in phenotyping technologies and their application in field studies have enabled detailed measurements of complex traits across time and space (e.g., Bucksch et al., 2014; Landi et al., 2010; J. P. Lynch et al., 2014; Rajurkar et al., 2022; Shao et al., 2021; Woods et al., 2022). Historically, field studies have relied on manual scoring methods to assess root crown architecture, such as Shovelomics (Trachsel et al., 2011) and root pulling force (RPF) (Landi et al., 2002; Ortman et al., 1968; Woods et al., 2022). RPF quantifies the vertical force required to extract root crowns from the soil and has recently been scaled for higher throughput applications through mechanization (McKay et al., 2023). Semiautomated imaging and analysis pipelines, including optical two‐dimensional (2D), and optical and X‐ray‐based three‐dimensional (3D), have enhanced the data quality obtained from mature root crowns, providing insights into root morphology and function (Bray & Topp, 2018; Das et al., 2015; Jiang et al., 2019; S. Liu et al., 2021; Lobet, 2017). However, these methods present trade‐offs between capturing phenotypic variation under agronomically relevant conditions and obtaining high‐throughput detailed data (York & Lobet, 2017). For example, X‐ray tomography (XRT) provides detailed in situ or post‐extraction insights into plant structure‐function relationships that most other root phenotyping methods cannot achieve; however, its application is limited by higher resource costs and lower throughput (Duncan et al., 2019; Helliwell et al., 2017; Ju et al., 2024; Mooney et al., 2012; Zeng et al., 2021). While numerous methods exist for measuring RSA, no single technique captures the full phenotypic complexity of the root crown architecture. Therefore, we hypothesize that integrating multiple phenotyping technologies can improve genotype–phenotype mapping in field studies of maize root crowns, enabling more robust analyses of complex traits in agronomically relevant conditions.

RSA is a complex trait controlled by numerous genetic and environmental factors (Cai et al., 2012; Champoux et al., 1995; Rogers & Benfey, 2015; Stuber, 1995). In rice, two genes have been identified as major contributors to phenotypic variation in RSA: *DEEPER ROOTING 1* (*DRO1*; Uga et al., 2013), which regulates root gravitropism, resulting in steeper growth angles and deeper root systems characteristic of the “steep, cheap, and deep” (SCD; J. P. Lynch, 2013) ideotype, and *Phosphorus‐Starvation Tolerance 1* (Gamuyao et al., 2012), which promotes early crown root formation under low phosphorus conditions. In maize, several genes influencing abnormalities in root development have been cloned; however, these studies have largely focused on seedlings, and their effects on mature root systems remain poorly understood (reviewed thoroughly by Feix et al., 2002 and Hochholdinger et al., 2018). While numerous RSA QTLs have been identified in mature maize through field studies, only a small number of these have been cloned. These include *Rootless1* (*Rt1*), which has a large effect on shoot‐borne and lateral root development across growth stages (Jenkins, 1930), as well as the root‐expressed *CBL‐interacting serine/threonine‐protein kinase 15* (*ZmCIPK15*; Schneider et al., 2022) and auxin‐related genes *ZmRSA3.1* and *ZmRSA3.2* (Ren et al., 2022), which regulate rooting angle and depth. This study builds upon previous findings by providing detailed field annotations of mutant phenotypes in mature maize root crowns. Specifically, we examine allelic variants of *DRO1* (“*dro1‐1*”) and *Rt1* (“*rt1‐2*”; Ju et al., 2024).

Bridging the gap between the identified RSA QTLs and functionally validated genes from field studies remains challenging, primarily because of the difficulty in capturing the full phenotypic space. In this study, we systematically evaluated phenome‐to‐genome variation in mature maize root crowns to assess how different field phenotyping approaches quantitatively capture RSA and to advance our understanding of its genetic architecture through genome‐wide and single‐gene analyses. To achieve this, we employed a diverse set of phenotyping approaches in three independent field experiments, including manual measurements of root biomass and RPF, as well as measurements of RSA obtained through 2D and 3D imaging and feature extraction. We hypothesized that employing multi‐view 2D and 3D imaging techniques will enhance the resolution and accuracy of extracting RSA information from field‐excavated root crowns, thereby improving the detection and characterization of key RSA QTL. Through this integrative approach, we aimed to provide insights into the use of high‐throughput root phenotyping technologies to survey root crown architecture for future field studies.

**Core Ideas**

- Complementary phenotyping approaches are powerful for studying the genetic basis of root system architecture.
- Multi‐view imaging enhances root trait estimation in two‐dimensional root crown phenotyping for genotype–phenotype mapping.
- Multivariate approaches effectively capture complex root traits and their underlying genetic architecture.
- Three‐dimensional phenotyping offers detailed insights to root crown analysis for forward and reverse genetic studies.
- Root pulling force is highly heritable and cost‐effective for large‐scale root crown field phenotyping.

## 2 MATERIALS AND METHODS

### 2.1 Germplasm and experimental design

We investigated the genetic basis of RSA phenotypes across three field experiments conducted at the Colorado State University Agricultural Research Development and Education Center in Fort Collins, CO (40.649°N, −105.000°W) during the summers of 2019, 2022, and 2023.

In the first experiment (2019), we evaluated quantitative variation in root traits measured in 312 inbred lines from the shoot apical meristem (SAM) maize diversity panel (Leiboff et al., 2015). Seeds were sown on 14 May using a split‐plot design with two treatment levels of factor irrigation: full irrigation (wet) or limited irrigation (dry), each consisting of three field replicates per genotype. Roots were sampled in both irrigation treatments when approximately 30% of the lines reached anthesis, hereafter referred to as “mid‐flowering” at 13 weeks post‐planting. Details of the field experiment have been described previously (Woods et al., 2022).

In the second experiment (2022), we employed a maize population of recombinant inbred lines (RILs) that segregated for root trait variations to compare RSA heritability estimates derived from 2D single‐ and multi‐view imaging and analysis of maize root crowns. The RIL population was obtained from a cross between two nested association mapping (NAM) parental lines, B73 and CML69, as described by Buckler et al. (2009). Based on soil nutrient analysis, the field was fertilized to recommendations for 200 bu/ac yield, amounting to 53.9 kg/ha monoammonium phosphate and 401.4 kg/ha urea prior to planting. Lines were planted in two‐row, 6.1‐m plots, with 76‐cm row spacing and 22‐cm seed spacing. Seeds were sown on May 19 in duplicate blocks under full irrigation, averaging approximately 2.5 cm per week. These root crowns were sampled prior to anthesis at 11 weeks post‐planting.

In the third experiment (2023), two maize root development mutants and their respective wild‐type allele lines were used to evaluate the effects of single‐gene mutations on quantitative RSA phenotypes. The *dro1‐1* allele, a *Mu* transposon insertion in *DRO1*, was introgressed into the W22 genetic background through a single generation of backcrossing to reduce background mutations (Feng et al., 2022). Similarly, the *rt1‐2* allele, a *Ds* insertion in *Rt1*, was introgressed into the T43 genetic background through one generation of backcrossing (Ju et al., 2024). The resulting plants from each population were self‐pollinated for three generations and selected for mutant and wild‐type alleles homozygous for each gene. For wild‐type comparisons in the field, the original W22 inbred line provided the wild‐type control for *dro1‐1*, while a wild‐type segregant replaced the original T43 inbred as the control for *rt1‐2*. Prior to planting the experiment, soil samples were collected, and nutrient levels were quantified. The field was then fertilized according to the recommendations for a targeted yield of 200 bu/ac, amounting to 107.8 kg/ha monoammonium phosphate and 339.7 kg/ha urea. Planting occurred on May 24, with seeds sown at 22‐cm spacing in two‐row, 6.1‐m plots with a row spacing of 76 cm. Throughout the growing season, the plots received approximately 2.5 cm of weekly irrigation and were regularly weeded by hand. Phenotyping for root system size was conducted post‐anthesis at 21 weeks post‐planting.

### 2.2 Field phenotyping

In the 2019 field experiment, maize root crowns were extracted from soil using manual root pulls. During this process, the maximum force required to extract the root system from the ground was measured using handheld force gauges and designated as the RPF (Woods et al., 2022). The throughput of this method was improved in 2022, where root crown excavations were conducted using a custom root pulling device mounted onto a high clearance tractor (McKay et al., 2023). This mechanical device utilizes a vision system to identify maize stalks and securely grasps them by closing a gripper around each stalk and then vertically extracting the plants from the soil. Root systems in 2023 were shoveled out manually. The excavated root systems were washed with water and air‐dried by hanging, yielding a total sample size of *N*_SAM_ = 1546, *N*_RIL_ = 193, and *N*_MU/WT_ = 29 cleaned root crowns for 2019, 2022, and 2023, respectively. Root biomass measurements were taken across all three field experiments after the root crowns were air‐dried to a constant weight.

### 2.3 Root imaging and feature extraction

**FIGURE 1:**
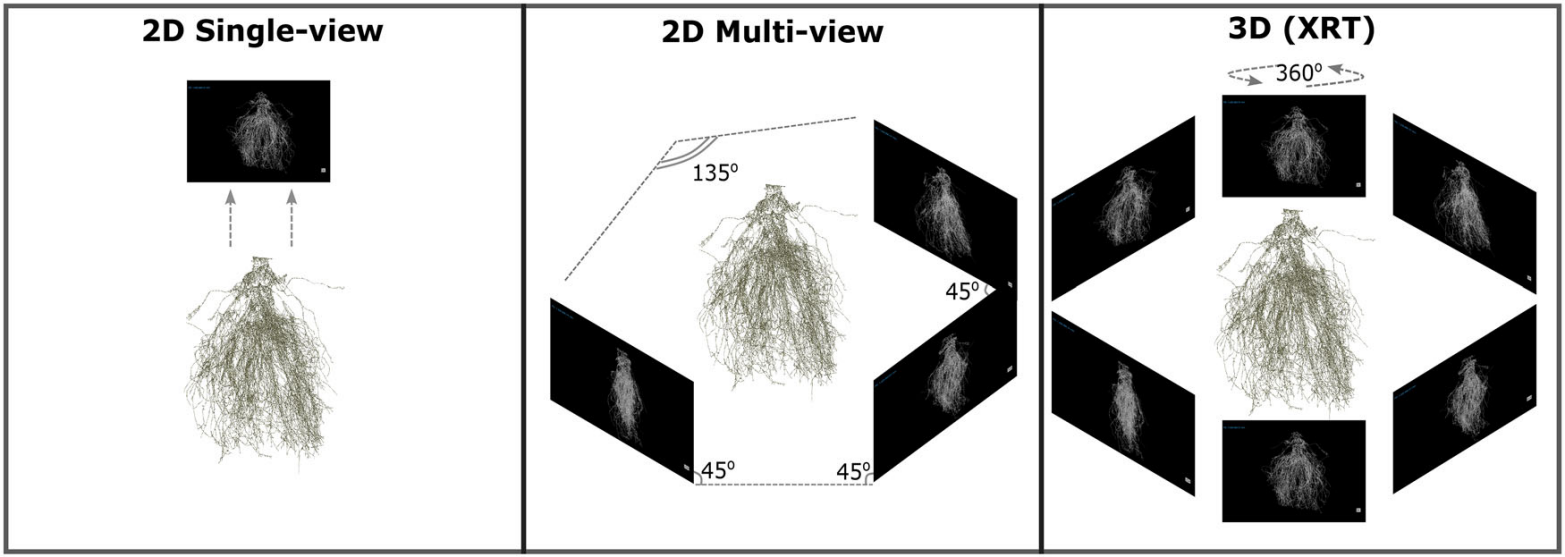
Simplified schematic of the relative amount of root crown data captured by each imaging platform. 2D, two‐dimensional; 3D, three‐dimensional; XRT, X‐ray computed tomography.

We performed image‐based analyses of the maize root systems to quantify and compare field‐grown variations in root architectural traits using three imaging pipelines (Figure 1). Note that 2D images were captured by suspending the root samples vertically in front of a dark background using a digital single‐lens reflex camera. These images were then processed and analyzed using the 2D digital imaging of root traits (DIRT) analysis software (Das et al., 2015). Note that 3D root imaging and analysis were conducted using XRT and a custom image processing and feature extraction pipeline (Donald Danforth Plant Science Center), as previously described (Shao et al., 2021), referred to here as the root crown analysis pipeline (RCAP). Detailed descriptions of RCAP trait implementations and related resources are available at: https://github.com/Topp‐Roots‐Lab/3d‐root‐crown‐analysis‐pipeline/. Finally, we reproduced a cost‐effective multi‐view imaging system, as described in Tovar et al. (2018), to simultaneously capture the features of root crowns from three angles (2D multi‐view). Raspberry Pi computers controlled the individual 6 MP cameras, which were positioned with a rotation around the root system of 45° for one camera pair and a 90° separation between the second and third cameras, resulting in 135° of rotation between the furthest pair of cameras (Figure 1). For detailed information and protocols regarding software and hardware implementation, refer to Tovar et al. (2018). The 2D multi‐view RSA measurements were obtained through 2D feature extraction of maize root crowns using DIRT image analysis software (Das et al., 2015) and were computed as the aggregate genotype averages based on three rotated viewpoints.

### 2.4 Descriptive statistics of phenotypic space

Statistical analyses were conducted using R version 4.3.0 (R Core Team, 2023) and JMP Pro 18 (JMP Statistical Discovery). To determine which RSA traits exhibited sufficient genetic variation for variant discovery in 2D phenotyping, we analyzed root crowns from the 2022 NAM RIL maize population. Traits derived from 2D single‐view and multi‐view imaging were tested for significantly nonzero genotypic variance components using a linear mixed model with genotype treated as a random effect. Significance was determined using a Wald test (*p *< 0.05). For traits showing a significant genotypic variance, broad‐sense heritability (*H* ^2^) was calculated as the proportion of genotypic variance to total phenotypic variance, and estimates were then compared between the single‐view and multi‐view approaches. To further assess the contribution of individual camera perspectives to the multi‐view results, heritability was also estimated separately for each of the three camera perspectives. These results were visualized using R/ggplot2 (Wickham, 2016).

In the 2019 experiment, we examined RSA in 312 inbred lines from the SAM maize diversity panel. Outliers within each treatment and genotype group were identified and excluded using the Bonferroni outlier test in R/car (Fox & Weisberg, 2019). Observations with Bonferroni‐adjusted *p*‐values < 0.05 were considered mean‐shift outliers. Trait heritability was estimated as the proportion of genotypic variance to total variance using a random effect one‐way analysis of variance model. Specifically, the phenotypic data were partitioned by treatment and then analyzed using the “lmer” function in R/lme4 (Bates et al., 2015), treating genotype as a random effect in the model.

Genotype averages for the maize diversity panel were calculated as best linear unbiased predictors (BLUPs), treating genotype as a random effect within treatments using the R/lme4 package (Bates et al., 2015). Spearman's rank genetic correlations were then computed from the BLUPs using R/cor (R Core Team, 2023). Principal component analysis (PCA) was performed on the remaining trait variables by employing the function “PCA” in R/FactoMineR (Lê et al., 2008). Axis selection was visually determined by analyzing a scree plot of the canonical eigenvalues. Clustering analyses were visualized using R/factoextra software (Kassambara & Mundt, 2020).

### 2.5 Quantitative genetic control of RSA in SAM maize genotypes

We implemented a genome‐wide association study (GWAS) on 312 SAM lines to identify genetic variation associated with root phenotypes and assess how different methods of quantifying RSA captured this variation. Prior to analysis, the phenotypic data were partitioned by irrigation treatment, and BLUP values were subsequently calculated for each trait by treating genotype as a random effect using the “lmer” function in R/lme4 (Bates et al., 2015). These values were used as the univariate GWAS response variables. PCA was then used to generate multivariate RSA traits, referred to as “PCs,” using the scaled BLUP values from each set of imaging traits (2D single‐view and 3D) in R/prcomp (R Core Team, 2023). The data derived from the genotypes on the first two PCA axes were used as GWAS multivariate response variables in the subsequent analyses. These variables were denoted as “2D PC1,” “2D PC2,” “3D PC1,” and “3D PC2” for each environment.

GWAS was conducted on 115 univariate and four multivariate (PC) traits derived from BLUPs (see Table S1) under full and limited irrigation, using the fixed and random model circulating probability (FarmCPU) method in GAPIT (Lipka et al., 2012; X. Liu et al., 2016). The initial HapMap genotype matrix for the SAM panel contained 1.2 million single nucleotide polymorphisms (SNPs) (Leiboff et al., 2015), which was refined to 860,000 after imposing a minor allele frequency filter of 5%. The first three principal components (PCs) and a kinship matrix calculated using GAPIT were used as covariates to control for population structure. Only traits with *Q–Q* plots displaying *p*‐value distributions that approximated normality were included in the FarmCPU results. SNPs whose significance passed the Benjamini–Hochberg false discovery rate threshold (0.10) were further investigated to determine the presence of co‐located QTL across methods, including the list of 2019 RSA‐associated SNPs previously identified by Woods et al. (2022).

RSA‐associated SNPs were fitted as random effects to estimate the SNP‐attributed and residual variance components using the R package lme4 v1.1‐34 (Bates et al., 2015). The proportion of phenotypic variance explained (PVE) by each SNP was calculated as the ratio of their corresponding variance to the total variance. Candidate regions of SNP colocalization were identified using 1‐Mb bins within each chromosome. Bin indices were calculated based on the number of complete bins that occur before the current SNP position within each chromosome, using the following formula:

$$\mathrm{Bin}\;\mathrm{Inde}x_{\mathrm{Chromsome}}=\left|\frac{\mathrm{Position}\;-\;1}{1\;\mathrm{Mb}}\right|+1,$$

where “Position” represents the SNP position mapped to the v2 B73 reference genome, and the bin size in the denominator, set to 1 Mb, is specified in base pairs (bp). The results were visualized using R/ComplexHeatmap, R/factoextra, and R/VennDiagram (Chen, 2022; Gu, 2022; Gu et al., 2016; Kassambara & Mundt, 2020).

### 2.6 Evaluation of single‐gene perturbed maize genotypes

In the 2023 experiment, two maize root development mutants and their corresponding wild‐type allele lines were used to examine how 2D single‐view, 2D multi‐view, and 3D imaging approaches detect the effects of single‐gene mutations on individual RSA phenotypes and patterns of among‐trait covariances. Differences in average phenotype response between mutant and wild‐type alleles were assessed using a two‐sample *t*‐test in R/t.test (R Core Team, 2023), with a significance level (*α*) of 0.05.

To estimate mutational covariance in the maize mutant and wild‐type allele lines, we conducted a Pearson correlation analysis using R/cor.test (R Core Team, 2023) to calculate the genetic correlation between RSA phenotypes within and among imaging methods. In addition, we applied a multivariate analysis of variance (MANOVA) to test for pairwise interactions among all trait combinations in individual models. We evaluated the results of the MANOVA models at a significance level (*α*) of 0.05.

## 3 RESULTS

### 3.1 Multi‐view 2D imaging enhances detection of heritable variation in maize root crown architecture under field conditions

**FIGURE 2:**
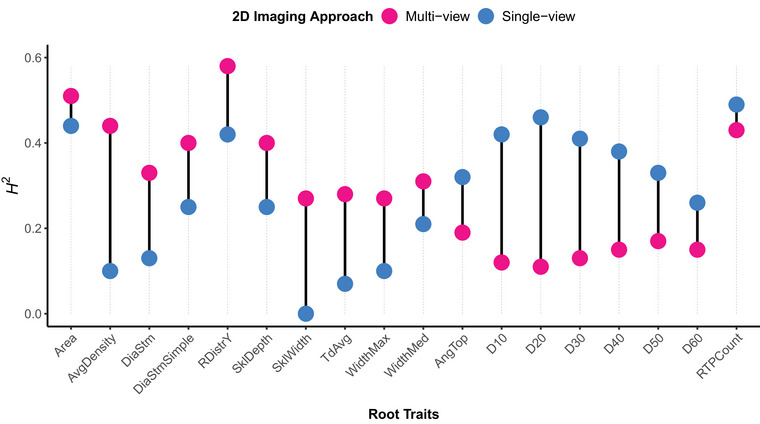
Comparison of broad‐sense heritability (*H* ^2^) between two‐dimensional (2D) single‐view and multi‐view approaches for root traits in the 2022 CML69 × B73 recombinant inbred line (RIL) population. Traits were extracted from root crown images using the DIRT (digital imaging of root traits) software (see Das et al., 2015, for a complete list of trait descriptions). The 18 traits shown were selected based on significant *H* ^2^ estimates in at least one method (Wald test, *p* < 0.05).

In the 2022 CML69 × B73 NAM RIL population, 18 of the 33 RSA traits showed significant genotypic variance (Wald test, *p* < 0.05) in at least one of the 2D phenotyping approaches. Heritability estimates were generally higher under the multi‐view setup, which also identified a broader range of significant traits, including those associated with root crown area, density, depth, and width (Figure 2; Table S2). In contrast, half of the traits detected by the single‐view approach were limited to depth‐derived traits, including accumulated width at 10%–60% depth (2D D10‐D60). To better understand the contribution of multiple viewpoints to the multi‐view approach, we compared heritability estimates from each individual camera perspective to those derived from the aggregated multi‐view trait values. The aggregated values used in the 2D multi‐view approach consistently provided better estimates than the individual camera perspectives from that setup, indicating added value for field‐driven RSA variant discovery pipelines by integrating multiple root crown perspectives (Table S3). Overall, the 2D multi‐view imaging approach captured a larger proportion of genetic variation underlying a broader array of geometrical and topological root crown traits compared to the single‐view approach.

### 3.2 Belowground phenotypic variation is largely driven by root system size and spread

We found a broad distribution of genotypic correlations among the different phenotyping methods in the 2019 SAM maize diversity panel (Figure S1). RPF showed the greatest positive correlations (*r* ≤ 0.70) with traits associated with overall root system size, including root biomass, 2D Area, 3D Volume, and fractal dimension side‐view (3D FractalDimensionS). These results were consistent with the findings of Shao et al. (2021) and Woods et al. (2022). Groups of related traits (i.e., those with multiple measurements) were both visually and statistically evaluated for removal based on weak overall correlations (*|r|* ≤ 0.30) and low variability (SD < 0.05) in relationships with traits measured using other phenotyping approaches (see Figure S1). For individual traits, we balanced the statistical considerations with biological relevance to maintain the integrity and interpretability of the analysis. The removal process aimed to minimize redundancy while retaining traits that captured unique variation or meaningfully contributed to the relationships between phenotyping approaches, resulting in the selection of 115 RSA traits for downstream analysis.

**FIGURE 3:**
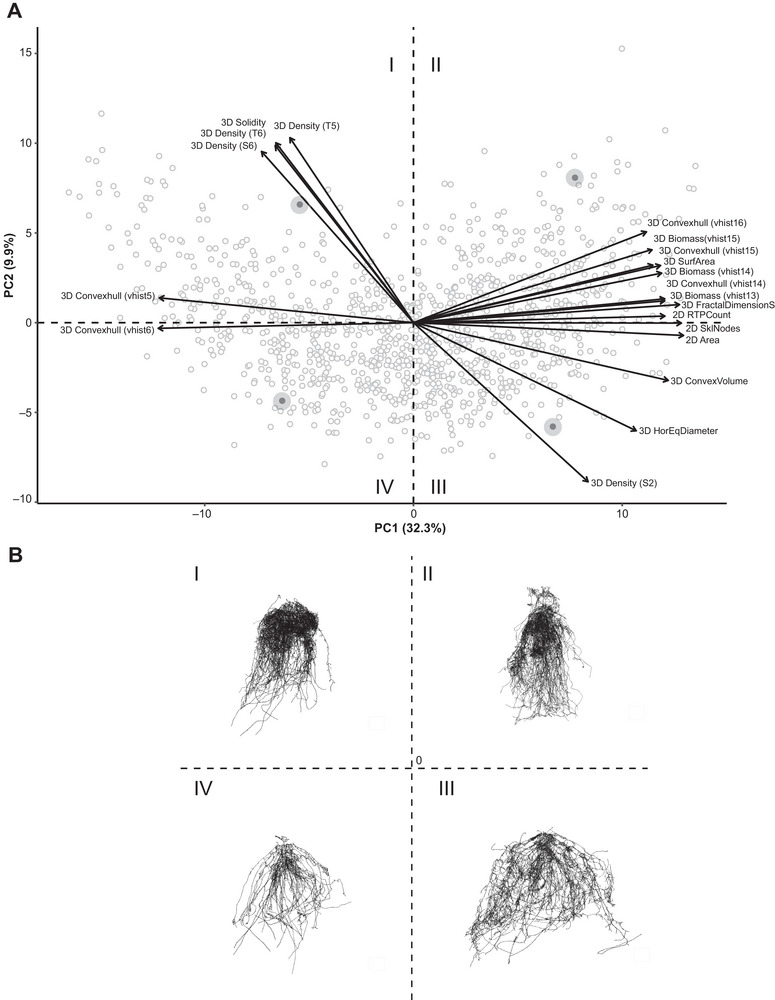
Relationship of 115 root system architecture (RSA) traits across 312 genotypes from the 2019 shoot apical meristem (SAM) maize panel. (A) Principal component analysis (PCA) biplot of the scaled first two principal components (PCs) for roots excavated at mid‐flowering. Open circles represent individual genotypes, and vectors indicate the top 20 RSA traits with the highest loadings on PC1 and PC2. Highlighted individuals are further illustrated in panel (B). (B) Comparison of maize root crown features through X‐ray computed tomography (XRT) scans of four distinct genotypes, contrasting the physical attributes of genotypes collected of the same replicate from each quadrant (I–IV) in the biplot. 2D, two‐dimensional; 3D, three‐dimensional.

PCA was used to investigate the phenotypic relationships among the remaining 115 RSA traits (Table S4). The first two PCs accounted for 32.30% and 9.90% of the total variation, respectively (Figure S2), with no discrete separation among individuals between treatment groups (Figure S3). We found a clear separation between the types of RSA traits that were strongly loaded on PC1 compared to PC2 (Figure 3A). PC1 predominantly captured variation in overall root system size and distribution, with higher scores positively correlated with 2D Area, 3D ConvexVolume, and 3D biomass distributions (vhist 13–16), indicative of larger root sizes. In contrast, PC2 captured differences in root density (3D Density S2, S6, T2, T4–T6), and in maximum root width (3D HorEqDiameter). Further evidence of these relationships was depicted by Figure 3B, which illustrated a sample of the variations in maize root crown size, shape, and distribution through XRT scans of four individuals from genotypes that were representative of each quadrant of the multidimensional space.

Estimates of *H* ^2^ in the SAM maize lines were generally higher for all evaluated RSA traits under wet conditions than under dry conditions (Figures S4 and S5; Table S5). This finding supports the prediction that low‐stress environments can increase the range of observable phenotypic variation, thereby facilitating clearer distinctions between genotypes (reviewed by Visscher et al. [2008]). Within the wet treatment, heritability estimates for 2D RSA traits highly correlated with PC1 ranged from 0.33 to 0.43, with 2D Area exhibiting the highest estimate. In comparison, 3D RSA traits associated with the same PC1 axis exhibited a broader distribution of heritability ranging from 0.19 to 0.45, with 3D FractalDimensionS showing the highest estimate. For traits associated with PC2, which primarily captured root density characteristics across 2D and 3D RSA traits, heritability ranged from 0.32 to 0.41, with the highest estimate observed for 3D Density T5. Although RPF and root biomass were not strongly correlated with PC1 and PC2, they demonstrated a higher average heritability than the traits obtained through 2D single‐view and 3D imaging (Figures S4 and S5).

### 3.3 Multivariate analysis identifies genome‐wide relationships among individual RSA traits

**FIGURE 4:**
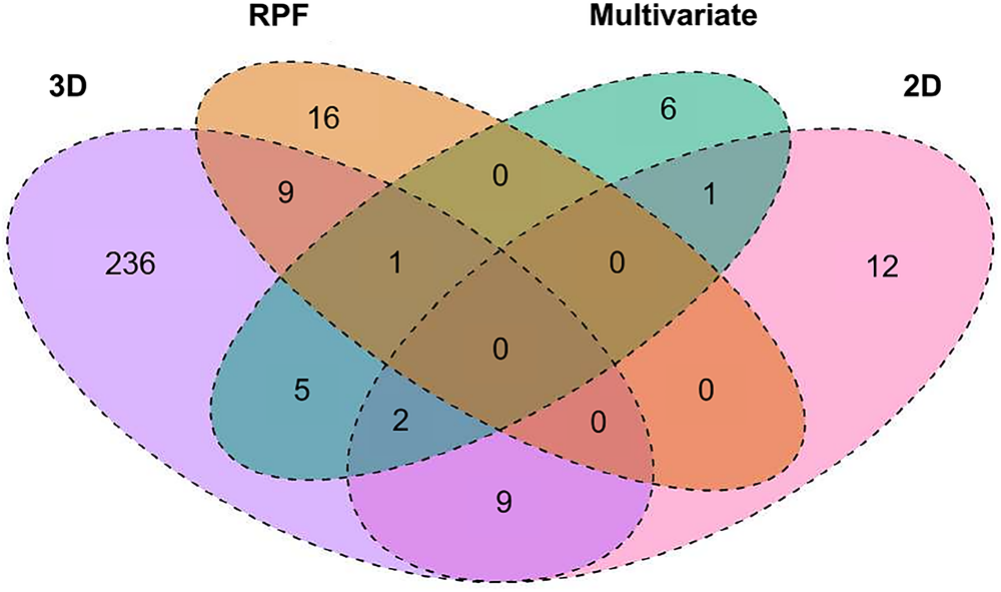
Venn diagram of genome‐wide association study (GWAS) single‐nucleotide polymorphism (SNP)‐associated 1‐Mb bins partitioned by phenotyping method. Overlapping regions indicate 1‐Mb bins that were enriched in two or more comparisons. 2D, two‐dimensional; 3D, three‐dimensional; RPF, root pulling force.

The FarmCPU GWAS models identified 405 significant SNPs across 288 distinct 1‐Mb bins associated with multivariate (PC) traits and univariate BLUP traits, including 2D single‐view, 3D, and RPF measurements. These findings build on the 22 SNPs previously reported by Woods et al. (2022), resulting in a cumulative total of 427 significant SNPs across 297 distinct 1‐Mb bins (Figure 4; Table S6). Incorporating both multivariate and univariate RSA traits as response variables in the GWAS models enabled better detection of trait‐specific and pleiotropic genomic signals. The combined set of significant SNPs were predominantly identified by 3D traits (83.84%), with 3D biomass distributions and 3D convex hull distributions accounting for the majority of these associations. This was followed by the RPF (7.03%), 2D (single‐view; 5.62%), and PC (3.51%) traits. A greater proportion of SNPs associated with individual RSA traits corresponded to RPF (7.03%), 3D Solidity (4.45%), and 3D Density S6 (3.75%). Notably, RPF shared the largest proportion of 1‐Mb bins with SNPs associated with other traits. RPF measurements were moderately heritable in both environments (wet treatment: *H* ^2^ = 0.35; dry treatment: *H* ^2^ = 0.49) and predominantly colocalized with 3D traits that had lower heritability. Specifically, these SNPs were associated with traits representative of root system density, and biomass and convex hull distributions within the upper 7 cm of the root crown, which cumulatively displayed higher values of PVE than RPF (Figure S6). Overall, 3D RSA traits accounted for the largest proportion of phenotypic variation and its corresponding genetic loci.

**FIGURE 5:**
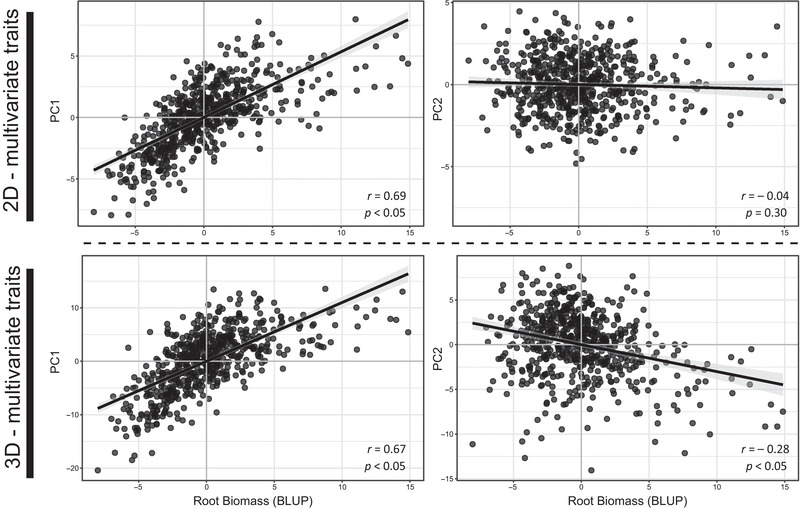
Correlation between genotypic values (best linear unbiased predictors [BLUPs]) of two‐dimensional (2D) and three‐dimensional (3D) genome‐wide association study (GWAS) multivariate root traits (PC1 and PC2) and root biomass in the 2019 shoot apical meristem (SAM) maize panel. *p*, probability value; *r*, Spearman's rank correlation coefficient. BLUP, best linear unbiased predictor; PC, principal component.

The use of multivariate RSA traits was instrumental in investigating key phenotype‐to‐genotype relationships. Approximately 60.0% (nine SNPs) of the SNPs associated with the multivariate traits were co‐located with 19 univariate traits. PC1 and PC2 explained substantial phenotypic variation, accounting for approximately 51% of the cumulative variation in 2D multivariate traits and 32% in 3D multivariate traits under both treatments. We decomposed the trait space of the eight multivariate traits to identify phenotypic relationships among genotypes along each principal axis (Table S7). Among the 2D multivariate traits, 2D PC1 primarily captured variations in root size and spatial characteristics, such as root area and lateral extent, while 2D PC2 largely described variations in root angle and depth. For the 3D multivariate traits, 3D PC1 represented variations in root distribution, including 3D biomass and convex hull distributions in the lower 50% (vhist 13–16) of the root crown, while 3D PC2 described variations in root density and uniformity. These relationships were captured on the genome‐wide level as SNPs associated with 2D PC1 were identified in multiple 1‐Mb bins that contained SNPs associated with individual 2D single‐view and 3D root density, area, and volume‐related traits. Similarly, the SNPs identified by 3D PC2 colocalized with individual 3D density and solidity traits (Figure S6). We estimated the phenotypic correlations between the multivariate traits and the breeding values for root biomass to obtain a biological ground truth for data interpretation and found significant positive correlations for root biomass with 2D PC1 (*r* = 0.69; *p *< 0.05) and 3D PC1 (*r* = 0.67; *p *< 0.05). In contrast, weaker correlations were observed with 2D PC2 (*r* = −0.04; *p *= 0.30) and 3D PC2 (*r* = −0.28; *p *< 0.05) (Figure 5). These results indicate that the first PC axes for both 2D and 3D RSA traits were strong predictors of belowground biomass.

### 3.4 Two‐dimensional multi‐view and 3D root phenotyping improve resolution in detecting phenotypic effects of two known‐RSA genes in mature maize root crowns

*Rt1* is a classic maize mutation known to reduce the number of lateral and nodal roots (Hochholdinger, 2009; Jenkins, 1930; Taramino & Sakai, 2009). However, the *rt1‐2* mutant allele has been shown to induce supernumerary nodal roots near the soil line, contrasting with the original *Rt1* phenotype and resulting in changes to the overall root crown architecture that resemble characteristics of the SCD ideotype (Ju et al., 2024; A. Liu et al., 2023; J. P. Lynch, 2013). In the 2023 field experiment, all three imaging approaches consistently captured the predicted geometrical root crown phenotypes for each allele. The *rt1‐2* mutation was associated with increased root growth angles and significantly reduced root system area and width‐to‐depth ratios (*p* ≤ 0.05) compared to wild‐type plants. However, significant differences in genotype averages for root system width were only observed in root crown features extracted from 2D single‐view and 3D imaging (Table 1).

**TABLE 1:** Statistical comparison of average root system architecture (RSA) traits between *dro1‐1* and *rt1‐2* maize mutant lines and their respective wild‐type allele lines across imaging approaches. Bold values denote statistical significance at the *p* ≤ 0.05 level.

*dro1‐1*						
Imaging	Trait	Wt	Mu	*df*	*t*	*p*
2D single‐view^a^	Area	8568.94	10,315.35	8	−1.69	0.13
	SklWidth	172.33	186.30	17	−0.82	0.42
	RTPCount	450.28	555.83	17	−1.48	0.16
	AngTop	57.13	45.20	10	2.53	**0.03** ^*^
	AvgDensity	2.05	3.81	6	−2.58	**0.04** ^*^
2D multi‐view^a^	Area	8568.95	10,315.34	8	−1.69	0.13
	SklWidth	161.12	184.03	17	−2.06	**0.05** ^*^
	RTPCount	111.14	145.22	15	−2.70	**0.02** ^*^
	AngTop	37.01	41.08	17	−0.81	0.43
	AvgDensity	7.66	8.93	14	−0.94	0.36
3D (XRT)^b^	HorEqDiameter	407.81	521.08	8	−3.39	**≤0.01** ^*^
	WDRatio	0.48	0.66	6	−2.72	**0.03** ^*^
	TotalLength	92,214.50	117,418.30	9	−2.32	**0.04** ^*^
	LengthDistr	0.12	0.14	6	−0.35	0.74
	NumberTips	1672.43	2327.67	10	−2.82	**0.02** ^*^
	Density T2	0.09	0.10	15	−2.07	**0.05** ^*^

*rt1‐2*						
Imaging	Trait	Wt	Mu	*df*	*t*	*p*
2D single‐view^a^	Area	9748.62	6999.19	11	3.96	**≤0.01** ^*^
	SklWidth	168.61	125.04	10	2.32	**0.04** ^*^
	RTPCount	573.29	359.71	12	3.00	**≤0.01** ^*^
	AngTop	44.46	45.45	10	−0.17	0.87
	AvgDensity	3.21	3.10	11	0.14	0.89
2D multi‐view^a^	Area	14,492.11	10,649.19	10	2.94	**0.02** ^*^
	SklWidth	163.29	136.02	7	1.47	0.18
	RTPCount	102.88	60.17	10	5.19	**≤0.01** ^*^
	AngTop	37.59	44.00	10	−1.21	0.25
	AvgDensity	10.47	8.58	10	1.17	0.27
3D (XRT)^b^	HorEqDiameter	466.00	363.87	9	5.50	**≤0.01** ^*^
	WDRatio	0.50	0.40	9	5.12	**≤0.01** ^*^
	TotalLength	127,777.70	83,140.00	11	4.52	**≤0.01** ^*^
	LengthDistr	0.29	0.08	6	2.50	**0.05** ^*^
	NumberTips	2385.57	1225.00	11	6.32	**≤0.01** ^*^
	Density T2	0.09	0.09	11	0.01	0.99

*Note*: AngTop = upper root system angle (°); Area = root area (mm^2^); AvgDensity = average root system density (unitless); Density T2 = top‐view root system density (unitless); HorEqDiameter = maximum root model width (voxel); LengthDistr = ratio of root length in the upper one‐third to the lower two‐thirds of model; Mu = mutant; NumberTips = number of root tips; RTPCount = root tip count (count); SklWidth = skeleton width (mm); TotalLength = cumulative root length (voxel); WDRatio = width‐to‐depth ratio (unitless); Wt = wild‐type.

Abbreviations: 2D, two‐dimensional; 3D, three‐dimensional; RCAP, root crown analysis pipeline; XRT, X‐ray tomography.

^a^ Image analysis using DIRT (digital imaging of root traits).

^b^ Image analysis using RCAP.

* Welch's *t*‐test (*p* ≤ 0.05).

**FIGURE 6:**
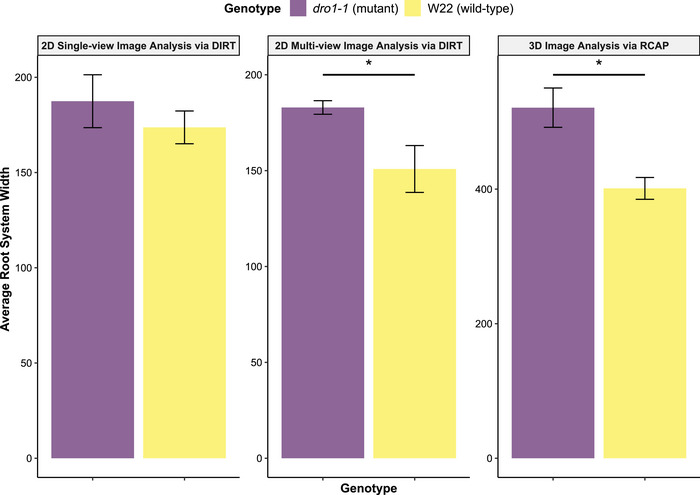
Genotype averages for root system width ± SE in *dro1‐1* mutant and wild‐type allele lines, measured across three imaging and analysis pipelines. Root system width was extracted as skeleton root width (two‐dimensional [2D] SklWidth) using DIRT (digital imaging of root traits) for both 2D single‐view and 2D multi‐view imaging approaches, and as maximum root system width (three‐dimensional [3D] HorEqDiameter) using root crown analysis pipeline (RCAP) for 3D imaging. Significant differences between genotypes are denoted by asterisks (*), with *p* ≤ 0.05.

Similarly, root crown imaging and digital phenotyping revealed significant variations (*p* ≤ 0.05) in root system size and horizontal spread between *dro1‐1* mutant and wild‐type allele lines (Table 1). Based on prior observations of the *DRO1* gene in maize (Feng et al., 2022) and rice (Uga et al., 2013), wild‐type plants were predicted to exhibit increased root gravitropism compared to mutant plants. As expected, the *dro1‐1* mutation was associated with significantly reduced root growth angles, as well as increased root system area and width‐to‐depth ratios compared to wild‐type plants (Table 1). In contrast to *rt1‐2*, both 2D multi‐view and 3D phenotyping improved genotype discrimination (*p* ≤ 0.05) for geometrical features in *dro1‐1* maize lines, such as root system width and number of root tips, which are typically constrained by the orthogonal positioning of the root crown in 2D single‐view imaging (Figure 6).

The analysis of mutational covariance of 3D RSA traits revealed detailed mutation‐specific relationships underlying differences in root gravitropism for the *dro1‐1* and *rt1‐2* maize lines. Two‐way MANOVA, accounting for genetic background, demonstrated significant genotype effects on 3D WDRatio and other traits, including total root length (3D TotalLength) (Pillai's Trace = 0.84, *F*(3, 33) = 7.18, *p *< 0.001) and root length distribution (3D LengthDistr) (Pillai's Trace = 0.81, F(3, 33) = 6.79, *p* < 0.001). In the *dro1‐1* lines, significant positive correlations were observed between 3D WDRatio and 3D TotalLength (*r *= 0.57; *p* = 0.009) as well as 3D LengthDistr (*r* = 0.73; *p *= 0.003). Similarly, *rt1‐2* lines exhibited significant positive correlations between 3D WDRatio and both 3D TotalLength (*r *= 0.73; *p* = 0.003) and 3D LengthDistr (*r* = 0.70; *p* = 0.005). The root crowns of both the *dro1‐1* wild‐type plants and the *rt1‐2* mutant plants displayed smaller width‐to‐depth ratios, reduced total root length, and a greater proportion of root length concentrated in the lower two‐thirds of the root systems. Overall, these results suggest that higher dimensional root phenotyping provides a more refined understanding of gene function and annotation of polymorphisms that influence root crown characteristics in the field.

## 4 DISCUSSION

The field of phenomics aims to enhance our understanding of genotype–phenotype maps through the systematic capture of whole‐organism phenotype information and multidimensional data analysis (Houle et al., 2010). Research in this domain has expanded the plant phenome and has identified new underlying gene models (Li et al., 2018, 2023; Ubbens et al., 2020). In this study, we evaluated multiple phenotyping and analytical frameworks for quantifying RSA in mature maize root crowns across three field experiments. Specifically, we assessed how different quantitative measurements of RSA describe the broader root crown phenome and determine the significance of this information for mapping genotype–phenotype relationships.

Overall, our results suggest that increasing the number of rotational viewpoints in 2D imaging using the 2D multi‐view approach enhances the capture of genetic variation controlling different aspects of maize RSA. In the 2019 SAM maize panel, phenome‐ and genome‐wide analyses indicated that 3D traits captured the largest proportion of phenotypic and genetic variation in field root crown architecture across different phenotyping methods. Among individual root traits, measurements of RPF were moderately heritable and strongly correlated with 3D traits, highlighting its potential as a cost‐effective alternative to XRT 3D imaging for large‐scale field mapping studies. Our findings also support the use of multivariate traits as an effective method for quantifying complex RSA phenotypes and identifying putative pleiotropic loci that are often missed by canonical univariate GWAS approaches. Analysis of the maize root development mutants, *dro1‐1* and *rt1‐2*, further validated the application of 3D and 2D multi‐view imaging within the context of a reverse genetics framework. Both methods detected statistically significant phenotypic variations that aligned with previous qualitative descriptions of RSA in their wild‐type and mutant allele lines—variations not captured by the 2D single‐view approach alone.

Trait‐based heritability estimates often vary based on measurement methods and environmental conditions (Hill et al., 1983; Hoffmann & Merilä, 1999; Macgregor et al., 2006). Our analysis of root crowns from the 2022 NAM RIL population demonstrated that integrating multiple viewpoints in 2D phenotyping enhances the detection of significant heritable variation in RSA under field conditions (Tables S2 and S3). As illustrated in Figure 2, the 2D multi‐view approach yielded relatively higher heritability estimates than the single‐view approach for various RSA features that broadly characterize root crown geometry and topology. For instance, 2D WidthMax, a direct measurement of maximum root system width (see Das et al., 2015), exhibited significant genotypic variance (*H* ^2^ = 0.27; *p *< 0.05) in the multi‐view setup, whereas it did not in the single‐view setup (*H* ^2^ = 0.10; *p *= 0.38) or in any other individual camera perspectives (Table S3). These findings were further supported by our analysis of the maize development mutants, where the multi‐view trait estimates provided better statistical power for detecting subtle differences in RSA, including root system width, between *dro1‐1* wild‐type and mutant plants (Table 1; Figure 6). For the *D*‐value traits (2D‐D60), where the 2D single‐view approach produced higher heritability estimates, these traits are associated with brace root orientation and root system spread and are derived from measurements of maximum root width, depth, and width at specific depth intervals (Bucksch et al., 2014). Similar to the *D*‐value traits, these associated traits showed differences among imaging perspectives (see Table S3).

Single‐view 2D imaging is a widely used and relatively straightforward approach to digital root phenotyping; however, it can introduce sampling bias to root feature extraction because root crowns are rarely symmetrical and are typically positioned with the widest part of the root system orthogonal to the camera (Tuberosa, 2012). These results suggest that in single‐view imaging, root crowns are often preferentially arranged to provide an orientation that optimally displays brace root orientation and root system spread, leading to higher end trait estimates. In contrast, the inclusion of additional perspectives in the multi‐view approach may summarize more variability around the sampled root crowns. Therefore, we recommend the 2D multi‐view approach as a readily implementable improvement to 2D single‐view for addressing forward and reverse genetic hypotheses, especially when investigating RSA traits that are greatly influenced by asymmetry and root crown orientation. Future research should focus on optimizing the integration of multi‐view image data into consolidated phenotypic values, as simple averages across images may fail to accurately capture the complexity of the field‐excavated root crowns.

Advances in 3D imaging technology have significantly enhanced our ability to interrogate root systems and capture the complexity of RSA in agricultural systems (Bohn et al., 2006; Dowd et al., 2022; Jung & McCouch, 2013; Li et al., 2023; S. Liu et al., 2021; Mattupalli et al., 2018; Morris et al., 2017; Shao et al., 2021; Topp et al., 2013). Our study reinforced this progress by showing that 3D measurements not only provided detailed insights at the phenome level but also had a greater capacity to detect significant variations in both genome‐wide and single‐gene analyses. PCA of SAM maize lines revealed substantial phenotypic variation driven by 3D traits, particularly those linked to root size, distribution, and density (Figure 3A). XRT scans of maize root crowns further validated these results, with root models visually reflecting size variations along PC1 and density variations along PC2, consistent with the vectors in the PCA biplot (Figure 3B). Furthermore, genome‐wide association analyses identified the largest number of RSA‐associated loci using 3D traits, with these loci showing higher average PVE values compared to those identified with 2D, RPF, and multivariate traits (Figure 4; Figure S6). Analyses of mutant and wild‐type allele lines for the *dro1‐1* and *rt1‐2* RSA genes provided evidence for the ability of 3D phenotyping to capture fine‐scale root allocation trade‐offs, consistent with previously reported qualitative descriptions (Figure 6; Table 1) (Feng et al., 2022; Hochholdinger et al., 2018; Jenkins, 1930; Uga et al., 2013). Overall, these findings underscore the value of 3D phenotyping as a powerful tool for capturing complex root system dynamics and advancing our understanding of genetic contributions to RSA in field studies of maize.

Although 3D phenotyping offers substantial advantages for genome‐wide studies of RSA in maize, its widespread application is often constrained by its associated overhead costs and training. In contrast, RPF measurements, which demonstrated high heritability and strong correlations with 3D traits, may serve as a more cost‐effective method for quantifying RSA for large‐scale field phenotyping (Shao et al., 2021; Woods et al., 2022). However, the suitability of its application varies depending on the system, root archetype, and experimental design. Notably, genome‐wide association analyses revealed numerous SNPs associated with lower heritable 3D traits that co‐located with RPF and explained a higher proportion of phenotypic variance for each bin (Figure S6). Based on the quantitative genetic theory, we hypothesized that the genetic architecture of RPF may be more polygenic in nature, with many genes contributing to small effects (Fisher, 1919, 1930). In contrast, the larger PVE values found among information‐rich 3D traits could be controlled by fewer loci with larger effects. Genome‐wide associations with RPF could have also been confounded by nonadditive genetic variation, as estimates of *H* ^2^ reflect the total genetic variation comprising additive, dominance, and epistatic effects (M. Lynch & Walsh, 1998).

Complex phenotypes in biological organisms emerge from interactions and contributions of individual traits. A common approach to studying the genetic underpinnings of these phenotypes involves using data reduction techniques, such as PCs, to generate “composite traits” (Hotelling, 1933; Klei et al., 2008; Li et al., 2023; Rice et al., 2020; Topp et al., 2013). Our study utilized 2D and 3D multivariate traits derived from PCA to test for genome‐wide associations. We found that these traits, particularly the 2D PC1 and 3D PC1, had strong positive associations with root biomass accumulation (Figure 5). Additionally, the loci identified by multivariate traits were primarily located within regions that were also associated with individual traits (Figure 4). Notably, the combination of individual traits for these regions reflected both the correlated (e.g., 3D PC2 and 3D root density traits) and orthogonal (e.g., 2D PC1 and 2D AvgDensity) phenotypic relationships observed in the PCA (Figure 3A; Figure S6). One explanation for these patterns is that similar and distinct elements of maize root architecture are controlled by the same genes, which is indicative of a pleiotropic genetic architecture. Although multivariate traits do not directly provide biological insights into pleiotropic loci, they are advantageous for elucidating the relationships between low‐ and highly heritable traits at a given locus, which are often missed by univariate approaches (Rice et al., 2020). These results further demonstrate that the multivariate approach has the power to replicate univariate results, reinforcing its value in forward genetic studies (Rice et al., 2020; Topp et al., 2013).

Understanding the genetic and molecular mechanisms that regulate root system development and architecture is critical for enhancing plant resilience to environmental stress, improving resource use efficiency, and supporting ecosystem services such as soil stabilization and carbon sequestration. Historically, much of our understanding of this area has been limited to studies in controlled environments, focusing on seedlings or model systems. While the dicot model organism *Arabidopsis thaliana* has provided a foundation for understanding root development, it does not directly translate to the genetic architecture of monocot crops, such as maize, rice, and sorghum, which differ in the development and morphology of their root systems (Jung & McCouch, 2013; Péret et al., 2009; Petricka et al., 2012; Scheres et al., 1996; Tuberosa et al., 2003; Ueda et al., 2005). This has created a gap in our ability to study complex, mature root systems in crops grown under agronomic environments, primarily because of the lack of appropriate phenotyping tools and analytical methods. Our research addressed this by applying advanced RSA phenotyping frameworks to mature maize root crowns, revealing that capturing multidimensional data, such as through 2D multi‐view, 3D phenotyping, and multivariate approaches, significantly improves the precision of phenotype–genotype mapping. These findings underscore the importance of employing complementary approaches to fully explore the phenotype–genotype gap. However, it is worth noting that the relationship between phenotyping methods and their effectiveness is not necessarily static. As feature extraction technologies—such as AI‐based tools, topological data analysis, and advanced 3D graphics—continue to evolve, the potential for capturing more comprehensive and accurate root phenotypes will grow. Therefore, future research should not only build on current methods but also remain adaptable, integrating cutting‐edge technologies to further refine our understanding of the root phenome and its genetic drivers under diverse agricultural conditions.

## AUTHOR CONTRIBUTIONS

**Kirsten M. Hein**: Conceptualization; data curation; formal analysis; funding acquisition; investigation; methodology; project administration; validation; visualization; writing—original draft; writing—review and editing. **Alexander E. Liu**: Methodology; resources; writing—review and editing. **Jack L. Mullen**: Conceptualization; data curation; formal analysis; funding acquisition; investigation; methodology; supervision; validation; writing—review and editing. **Mon‐Ray Shao**: Methodology; resources; writing—review and editing. **Christopher N. Topp**: Conceptualization; data curation; funding acquisition; investigation; methodology; resources; supervision; writing—review and editing. **John K. McKay**: Conceptualization; data curation; funding acquisition; investigation; project administration; resources; supervision; writing—review and editing.

## CONFLICT OF INTEREST STATEMENT

The authors declare no conflicts of interest.

## Supporting information




**Supplemental Figure S1** Heatmap of pairwise genotypic correlations among 155 RSA traits grouped by collection method (2D single‐view imaging, 3D imaging, and manual measurements) in the 2019 SAM maize panel.
**Supplemental Figure S2** Percentage of explained variances by individual principal components associated with the PCA of 115 RSA traits measured in the 2019 SAM maize panel.
**Supplemental Figure S3** PCA of RSA traits partitioned by irrigation treatment measured in the 2019 SAM maize panel.
**Supplemental Figure S4** Distribution of broad‐sense heritability estimates of the 2019 SAM maize panel RSA traits among phenotyping methods and irrigation treatments.
**Supplemental Figure S5** Broad‐sense heritability estimates for root mass (g) and RPF (kg) across irrigation treatments in the 2019 SAM maize panel.
**Supplemental Figure S6** Heatmap of the percentage of phenotypic variance explained (PVE) by 1‐Mb genomic regions associated with traits colocalized across imaging, RPF, and multivariate approaches.

**Supplemental Table S1** Genotypic values (BLUPs) used in the 2019 FarmCPU GWAS on the SAM maize diversity panel, with RSA univariate and multivariate traits partitioned by phenotyping method.
**Supplemental Table S2** Comparison of broad‐sense heritability for all RSA traits between 2D single‐view and multi‐view root crown imaging in the 2022 NAM RIL population.
**Supplemental Table S3** Comparison of 18 RSA traits that showed significant broad‐sense heritability (H2) in at least one 2D imaging method in the 2022 NAM RIL population.
**Supplemental Table S4** 2019 PCA trait loadings associated with the axes represented in Figure 3.
**Supplemental Table S5** Broad‐sense heritability and variance estimates for RSA traits in the 2019 SAM maize panel.
**Supplemental Table S6** List of significant SNPs from the 2019 FarmCPU GWAS.
**Supplemental Table S7** Multivariate GWAS trait loadings by phenotyping method and irrigation treatment in the 2019 SAM maize diversity panel.

## Data Availability

Raw phenotypic metadata are available on the Dryad Digital Repository (https://doi.org/10.5061/dryad.z34tmpgq4, http://datadryad.org/share/HeNYoxNMdN_GrHMyZHFN3rUTN1UiG8OFhU-B107E7mM). Phenotypic values used in the 2019 genome‐wide association analysis, along with the identified significant RSA‐associated SNPs, are provided in Tables S1, S6, and S7.
