# Supplementary material for: Phenome‐to‐genome insights for evaluating root system architecture in field studies of maize
Source: Plant Genome. 2025 Aug 24;18(3):e70100. doi: 10.1002/tpg2.70100 (PMC12375851; doi:10.1002/tpg2.70100)
Supplement: Supplementary file 1 — Supplemental Figure S1 Heatmap of pairwise genotypic correlations among 155 RSA traits grouped by collection method (2D single‐view imaging, 3D imaging, and manual measurements) in the 2019 SAM maize panel. Supplemental Figure S2 Percentage of explained variances by individual principal components associated with the PCA of 115 RSA traits measured in the 2019 SAM maize panel. Supplemental Figure S3 PCA of RSA traits partitioned by irrigation treatment measured in the 2019 SAM maize panel. Supplemental Figure S4 Distribution of broad‐sense heritability estimates of the 2019 SAM maize panel RSA traits among phenotyping methods and irrigation treatments. Supplemental Figure S5 Broad‐sense heritability estimates for root mass (g) and RPF (kg) across irrigation treatments in the 2019 SAM maize panel. Supplemental Figure S6 Heatmap of the percentage of phenotypic variance explained (PVE) by 1‐Mb genomic regions associated with traits colocalized across imaging, RPF, and multivariate approaches. [file TPG2-18-e70100-s002.pdf]

Phenome-To-Genome Insights for Evaluating Root System Architecture in Field Studies of Maize

Kirsten M. Hein, Alexander E. Liu, Jack L. Mullen, Mon-Ray Shao, Christopher N. Topp, and John K. McKay

Supplemental Figures (Pages 2-5)

Supplemental Figure S1 Heatmap of pairwise genotypic correlations among 155 RSA traits grouped by collection method (2D single-view imaging, 3D imaging, and manual measurements) in the 2019 SAM maize panel. Correlations were calculated using the Spearman method, with colors representing the correlation coefficient (*r*), ranging from red (-1, negative correlation) to blue (+1, positive correlation). Gray squares indicate NA values.

Supplemental Figure S2 Percentage of explained variances by individual principal components associated with the PCA of 115 RSA traits measured in the 2019 SAM maize panel.

Supplemental Figure S3 PCA of RSA traits partitioned by irrigation treatment measured in the 2019 SAM maize panel.

Supplemental Figure S4 Distribution of broad-sense heritability estimates of the 2019 SAM maize panel RSA traits among phenotyping methods and irrigation treatments.

Supplemental Figure S5 Broad-sense heritability estimates for root mass (g) and RPF (kg) across irrigation treatments in the 2019 SAM maize panel.

Supplemental Figure S6 Heatmap of the percentage of phenotypic variance explained (PVE) by 1-Mb genomic regions associated with traits colocalized across imaging, RPF, and multivariate approaches. The color intensity reflects the percentage of PVE attributed to significant GWAS SNPs identified by this study for each 1-Mb region. The marginal plot above the heatmap displays the genomic distances between SNPs within each 1-Mb region, with error bars representing the minimum and maximum distances (in kb) and stars indicating the median distances.

Additional Data File 1 (Supplemental_Results.xlsx)

Supplemental Table S1 Genotypic values (BLUPs) used in the 2019 FarmCPU GWAS on the SAM maize diversity panel, with RSA univariate and multivariate traits partitioned by phenotyping method.

Supplemental Table S2 Comparison of broad-sense heritability for all RSA traits between 2D single-view and multi-view root crown imaging in the 2022 NAM RIL population.

Supplemental Table S3 Comparison of 18 RSA traits that showed significant broad-sense heritability (H2) in at least one 2D imaging method in the 2022 NAM RIL population. Estimates were derived from single-view and multi-view (averaged across three camera perspectives) imaging and feature extraction using the DIRT software. Camera-specific estimates from the multi-view set-up are also reported.

Supplemental Table S4 2019 PCA trait loadings associated with the axes represented in Figure 3.

Supplemental Table S5 Broad-sense heritability and variance estimates for RSA traits in the 2019 SAM maize panel.

Supplemental Table S6 List of significant SNPs from the 2019 FarmCPU GWAS.

Supplemental Table S7 Multivariate GWAS trait loadings by phenotyping method and irrigation treatment in the 2019 SAM maize diversity panel.

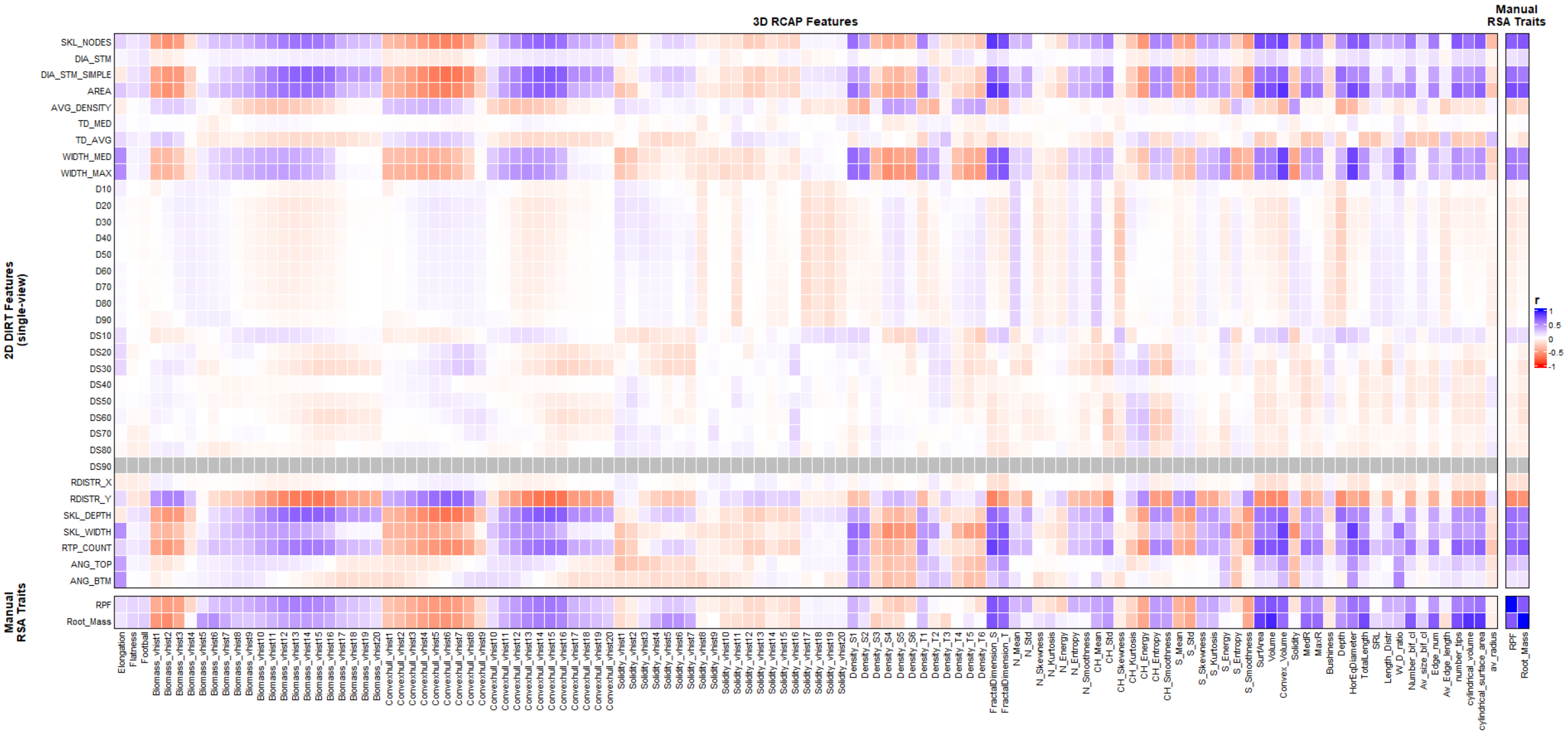

Supplemental Figure S1 Heatmap of pairwise genotypic correlations among 155 RSA traits grouped by collection method (2D single-view imaging, 3D imaging, and manual measurements) in the 2019 SAM maize panel. Correlations were calculated using the Spearman method, with colors representing the correlation coefficient (*r*), ranging from red (-1, negative correlation) to blue (+1, positive correlation). Gray squares indicate NA values.

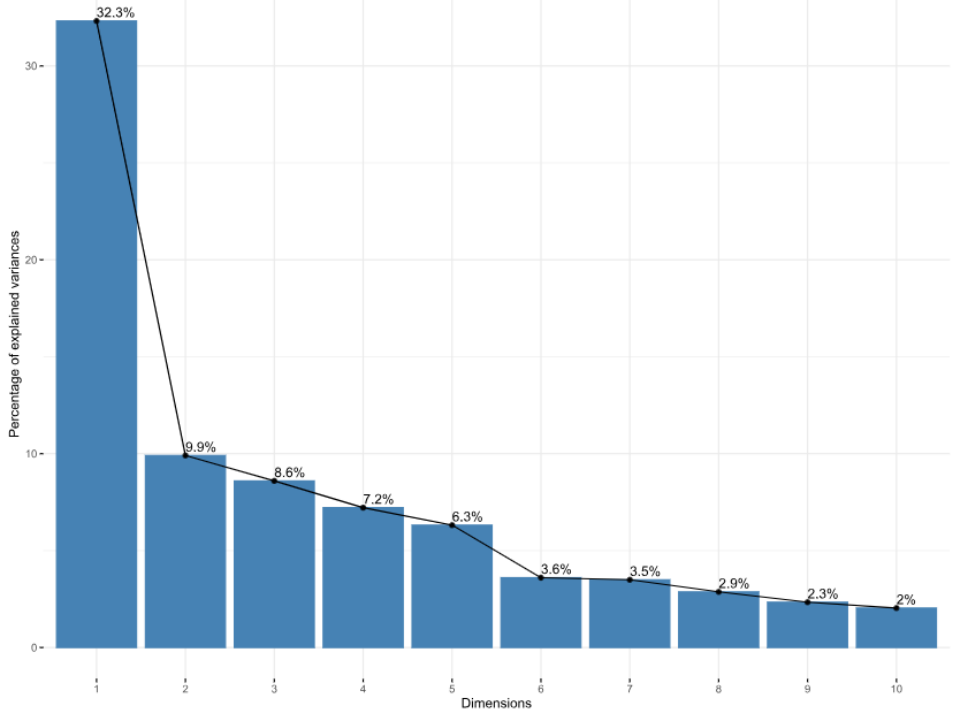

Supplemental Figure S2 Percentage of explained variances by individual principal components associated with the PCA of 115 RSA traits measured in the 2019 SAM maize panel.

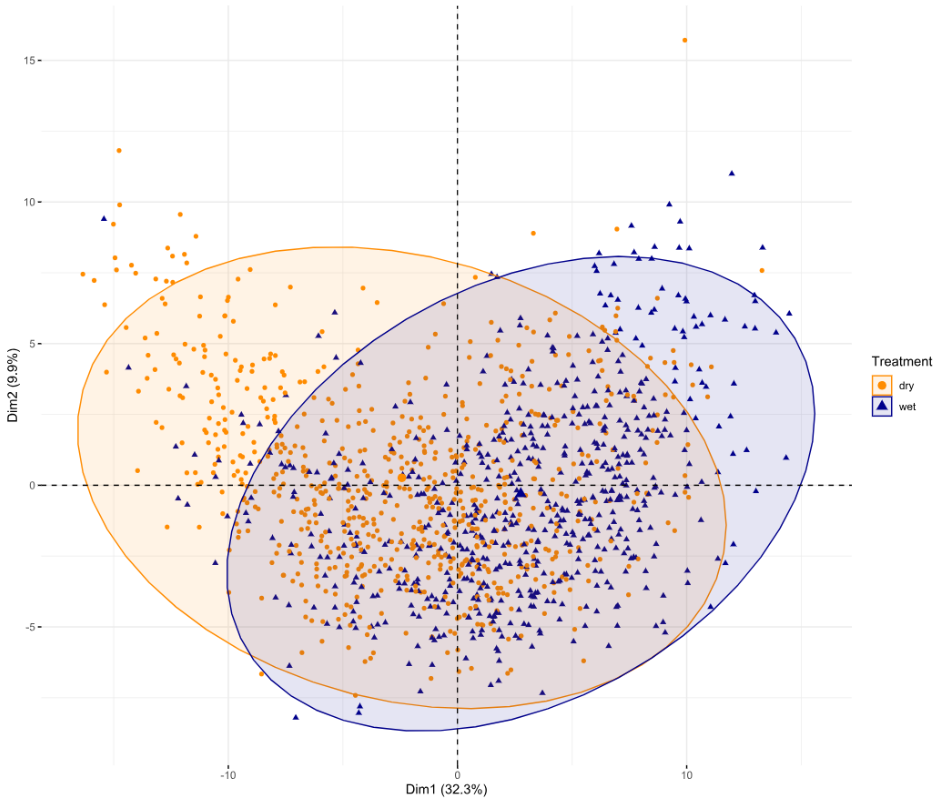

Supplemental Figure S3 PCA of RSA traits partitioned by irrigation treatment measured in the 2019 SAM maize panel.

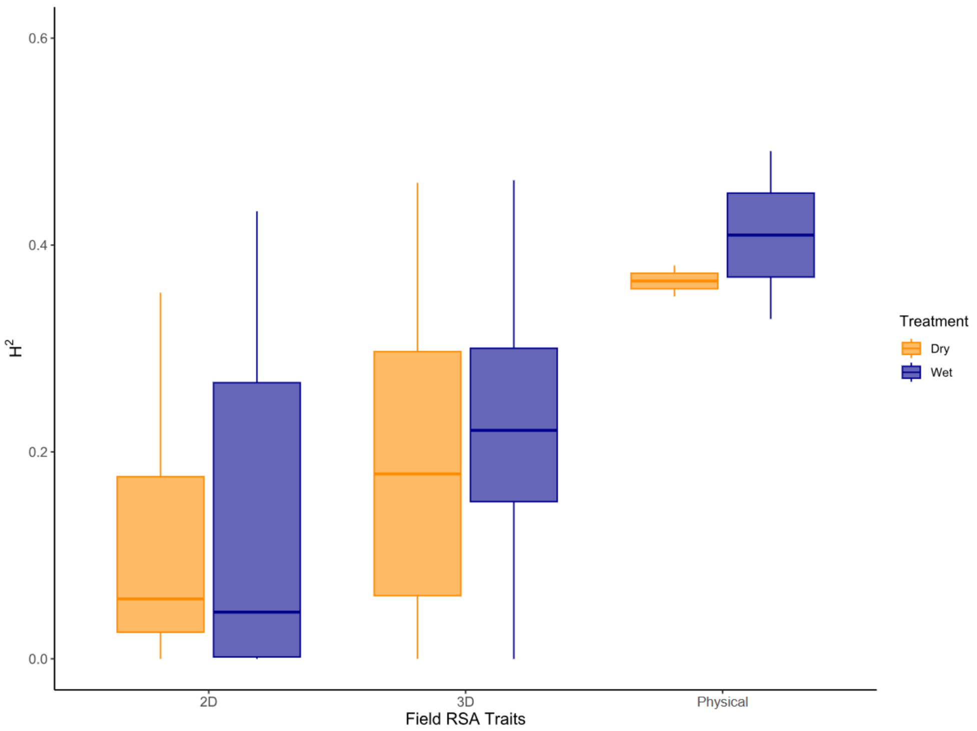

Supplemental Figure S4 Distribution of broad-sense heritability estimates of the 2019 SAM maize panel RSA traits among phenotyping methods and irrigation treatments.

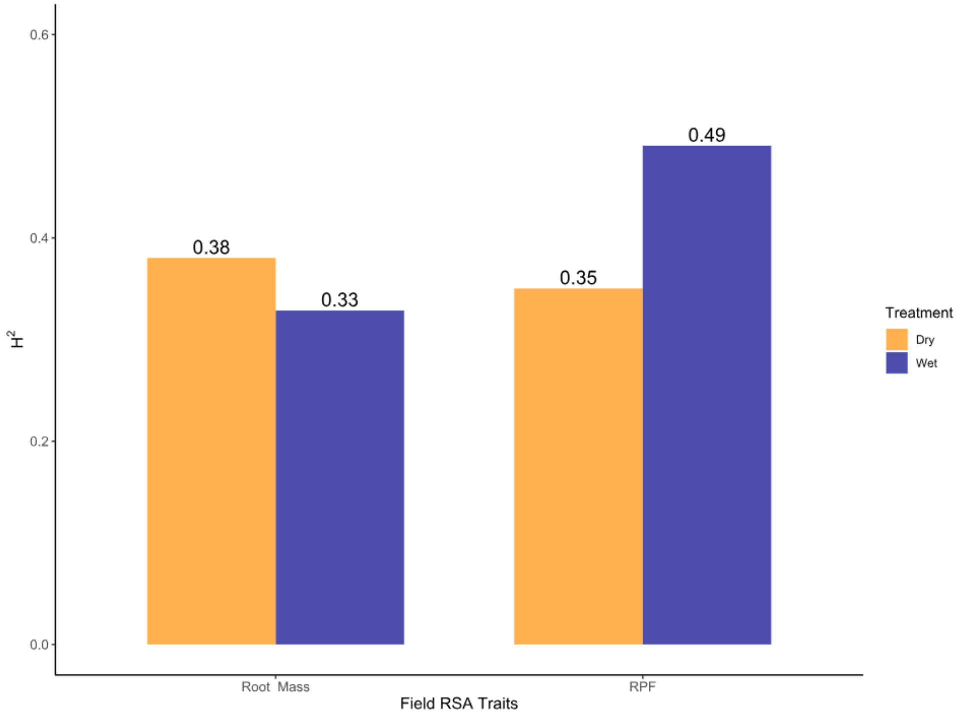

Supplemental Figure S5 Broad-sense heritability estimates for root mass (g) and RPF (kg) across irrigation treatments in the 2019 SAM maize panel.

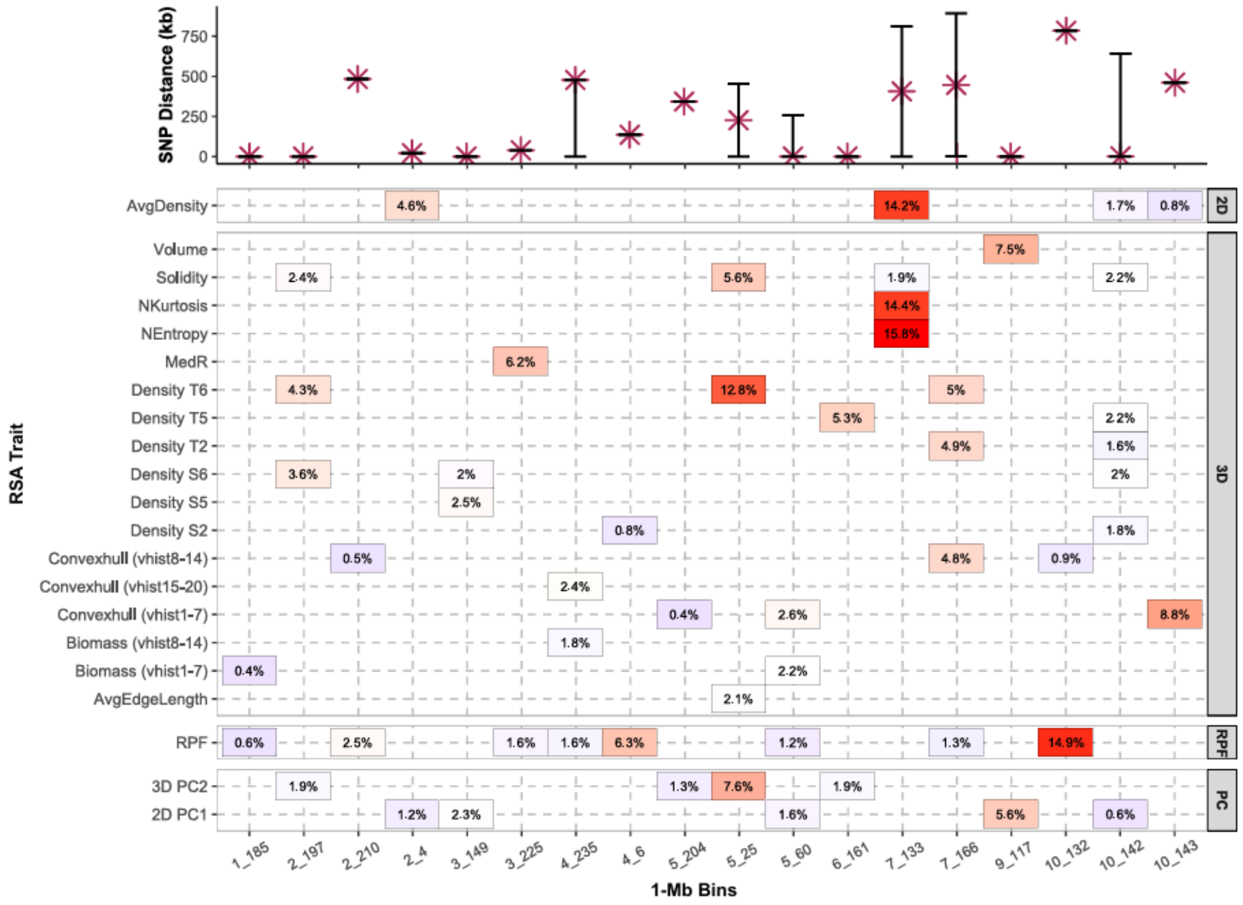

Supplemental Figure S6 Heatmap of the percentage of phenotypic variance explained (PVE) by 1-Mb genomic regions associated with traits colocalized across imaging, RPF, and multivariate approaches. The color intensity reflects the percentage of PVE attributed to significant GWAS SNPs identified by this study for each 1-Mb region. The marginal plot above the heatmap displays the genomic distances between SNPs within each 1-Mb region, with error bars representing the minimum and maximum distances (in kb) and stars indicating the median distances.
